# Lagged Effects of Exposure to Air Pollutants on the Risk of Pulmonary Tuberculosis in a Highly Polluted Region

**DOI:** 10.3390/ijerph19095752

**Published:** 2022-05-09

**Authors:** Yuqing Feng, Jing Wei, Maogui Hu, Chengdong Xu, Tao Li, Jinfeng Wang, Wei Chen

**Affiliations:** 1 State Key Laboratory of Resources and Environmental Information System, Institute of Geographical Sciences and Natural Resources Research, Chinese Academy of Sciences, Beijing 100101, China; fengyq.19s@igsnrr.ac.cn (Y.F.); xucd@lreis.ac.cn (C.X.); wangjf@lreis.ac.cn (J.W.); 2 College of Resources and Environment, University of Chinese Academy of Sciences, Beijing 100049, China; 3 Department of Atmospheric and Oceanic Science, Earth System Science Interdisciplinary Center, University of Maryland, College Park, MD 20740, USA; weijing_rs@163.com; 4 Jiangsu Center for Collaborative Innovation in Geographical Information Resource Development and Application, Nanjing 210023, China; 5 Chinese Center for Disease Control and Prevention, Beijing 102206, China; litao1@chinacdc.cn

**Keywords:** pulmonary tuberculosis, air pollution, epidemic characteristics

## Abstract

Background: Although significant correlations have been observed between air pollutants and the development of pulmonary tuberculosis (PTB) in many developed countries, data are scarce for developing and highly polluted regions. Method: A combined Poisson generalized linear regression–distributed lag nonlinear model was used to determine the associations between long-term exposure (2005–2017) to air pollutants and the risk of PTB in the Beijing–Tianjin–Hebei region. Results: The monthly PTB cases exhibited a fluctuating downward trend. For each 10 μg/m^3^ increase in concentration, the maximum lag-specific risk and cumulative relative risk (RR) were 1.011 (95% confidence interval (CI): 1.0091.012, lag: 3 months) and 1.042 (1.036–1.048, 5 months) for PM_2.5_, and 1.023 (1.015–1.031, 0 months) and 1.041 (1.026–1.055, 2 months) for NO_2_. The risk of PTB was negatively correlated with O_3_ exposure, and the minimum lag-specific risk and cumulative RR were 0.991 (95% CI: 0.987–0.994, lag: 0 months) and 0.974 (0.968–0.981, 4 months), respectively. No age-dependent effects were observed. Conclusions: Our results revealed potential associations between outdoor exposure to PM_2.5_, NO_2_, and O_3_ and the risk of PTB. Further research should explore the corresponding interactions and potential mechanisms.

## 1. Introduction

Tuberculosis (TB) is a respiratory infectious disease caused by *Mycobacterium tuberculosis*, which spreads when people who are sick with TB expel bacteria into the air, for example, by coughing. It remains one of the top 10 causes of death worldwide, despite a decline in incidence in recent years [1]. There were approximately 10 million new TB cases worldwide in 2019, and 1.4 million people died from the disease [2]. TB typically affects the lungs (pulmonary TB), but can also affect other sites (extrapulmonary TB). Approximately 25% of the global population is infected with *M. tuberculosis*; thus, these people are potentially at risk of developing TB [2]. Therefore, it is crucial to identify the predictors and risk factors of TB to prevent and control this disease. As risk factors of TB, smoking, diabetes, alcohol abuse, and indoor air pollution may reduce immunity and allow latent TB to develop into active TB [1].

Outdoor air pollution is also a risk factor for TB. Exposure to air pollution can directly affect respiratory organs and reduce lung function through increased pulmonary oxidative stress and persistent inflammation [3]. Li et al. revealed a potential association between outdoor exposure to PM_2.5_, PM_10_, SO_2_, and NO_2_ and active TB [4]. You et al. found that a 10 μg/m^3^ increase in the PM_2.5_ concentration during winter was significantly correlated with a 3% increase in the number of TB cases during the subsequent spring or summer in Beijing and Hong Kong [5].

Although many studies have been undertaken in developed countries and regions [6], the relationship between air pollution and pulmonary tuberculosis (PTB) in regions with high levels of air pollution has rarely been explored. Compared with other regions, areas with high levels of air pollution have different seasonal variations, latitudes, photoperiods, solar radiation, and other chronobiological conditions associated with PTB [7]. In addition, the composition and severity of air pollution also differ, leading to possible spatial heterogeneity in the relationship between air pollution and the lagged effects of TB. Pollutants that worsen air quality mainly include smoke, inhalable particles, SO_2_, and O_3_. China has set up more than 5000 monitoring stations at national, provincial, municipal, and county levels, and the ambient air quality monitoring network is now completed. The Beijing–Tianjin–Hebei region is one of the three largest city clusters in China. The region has a dense population and high levels of air pollution; thus, many people are exposed to air pollution that exceeds national standards and poses serious health hazards. The 2018 China Ecological and Environmental Bulletin, released by the Ministry of Ecology and Environment of China, indicated that 5 of the 13 cities in the Beijing–Tianjin–Hebei region were among the top 10 most polluted cities in China. The air quality index (AQI) quantitatively describes the air quality conditions, and the six main pollutants used for air quality evaluation are fine particulate matter (PM_2.5_), coarse PM (PM_10_), SO_2_, NO_2_, O_3_, and CO. For 338 Chinese cities, the average proportion of days in 2018 with “good” air quality was 79.3% (the number of days with an AQI of 0–100), whereas this was 50.5% for the Beijing–Tianjin–Hebei region. The primary pollutant is the pollutant with the highest individual AQI (IAQI) when the AQI exceeds 50. Among the six air pollutants included in the air quality evaluation, the number of days with PM_2.5_ as the primary pollutant accounted for the highest percentage of total exceedance days (AQI > 100), followed by O_3_, PM_10_, and NO_2_ [8].

Therefore, studies that assess the associations between exposure to air pollutants and TB risk in the Beijing–Tianjin–Hebei region can help fill the data gap for highly polluted regions. For the Beijing–Tianjin–Hebei region, this study aims to (i) describe the epidemic characteristics of PTB from 2004 to 2017, (ii) investigate the links between exposure to PM_2.5_, O_3_, and NO_2_ and the risk of PTB, and (iii) analyze the corresponding influence of age.

## 2. Material and Methods

### 2.1. Study Area

**Figure 1:**
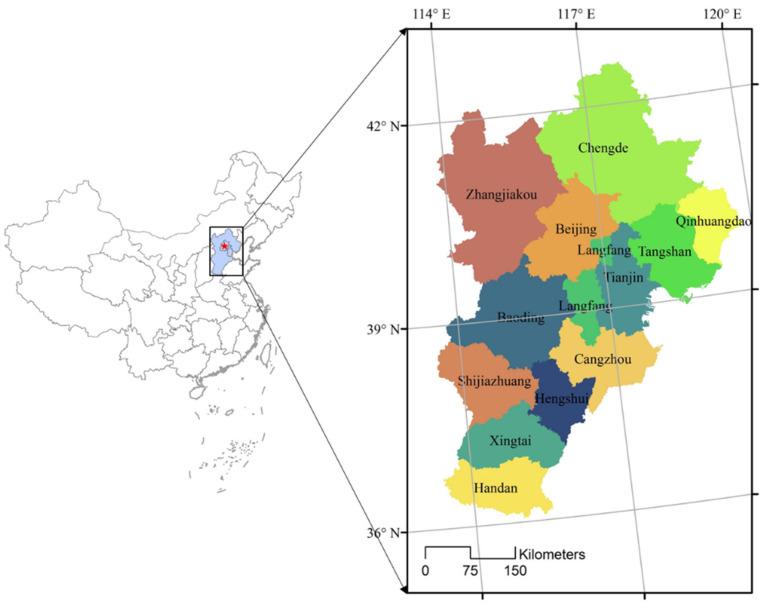
Geographical location of the Beijing–Tianjin–Hebei region.

The Beijing–Tianjin–Hebei region includes the Beijing and Tianjin municipalities and Hebei Province, which are primarily located on the North China Plain (Figure 1). The region has a total land area of 2 × 10^5^ km^2^, a population of >10^8^, and a continental temperate monsoon climate characterized by hot summers and cold winters.

The high levels of air pollution in the Beijing–Tianjin–Hebei region have attracted considerable attention in recent years. Pollution is largely caused by the large number of polluting industries in this region, such as cement, iron and steel, oil refining, and petrochemicals. Moreover, the local topography and climate are not conducive to pollution dispersion. In this study, we investigated three main air pollutants: PM_2.5_, O_3_, and NO_2_.

### 2.2. TB Cases

The number of notified PTB cases and the population of the Beijing–Tianjin–Hebei region from January 2005 to December 2017 were obtained from the China National Tuberculosis Information Management System (TBIMS). A total of 653,373 cases of PTB cases were identified.

The TBIMS was designed and developed by the China Center for Disease Control and Prevention (CDC) to collect TB-related information quickly, accurately, and completely, which played an important role in national TB control and prevention. TBIMS is a National Tuberculosis Program managed information system which includes all kinds of active TB, such as pulmonary TB (bacteriologically confirmed or clinically diagnosed) and extrapulmonary TB. A bacteriologically confirmed TB case is defined as a person with any positive result shown by sputum smear microscopy, culture or a WHO-approved nucleic acid amplification test. According to the national guidelines on TB control, all diagnosed TB (any kind) should be referred to TB designated health facilities and reported to TBIMS. All TB prevention and control facilities can record TB cases in real time on the system, which also synchronizes information on confirmed and suspected cases of TB reported by non-TB prevention and control facilities. Thus, it is possible to easily trace patients diagnosed by every hospital/clinic/primary health center throughout the country [9,10]. The basic demographic information, laboratory test results, diagnostic information, follow-up information, and treatment transfer results of every TB patient must be included in the system. The system includes four major functions: data collection, quality control, statistical analysis, and system maintenance. The data quality control module is used to ensure the timeliness, completeness, and accuracy of data entry [10]. In 2009, the TBMIS received a minor version upgrade, which included TB cases among the floating population and AIDS patients, as well as more information on multidrug-resistant cases.

### 2.3. Pollution and Meteorological Data

“ChinaHighPM_2.5_” and “ChinaHighO_3_” were collected from long-term, full-coverage, high-resolution, and high-quality datasets of ground-level air pollutants in China (ChinaHighAirPollutants). ChinaHighPM_2.5_ data were generated using MODIS/Terra + Aqua MAIAC aerosol optical depth products and other auxiliary data (e.g., ground-based measurements, satellite remote sensing products, atmospheric reanalysis, and model simulations). The monthly 0.01° (≈1 km) gridded ground-level PM_2.5_ products for the Beijing–Tianjin–Hebei region from 2000 to 2018 were used [11,12].

ChinaHighO_3_ data were similarly generated from big data using artificial intelligence by considering the spatiotemporal heterogeneity of air pollution [13]. Monthly Level-3 NO_2_ tropospheric VCD products are on a 0.25° × 0.25°grid, spatially aggregated from the Level-2 data, and included in the POMINO v2 dataset. The algorithm is based on the AMFv6 package called the LIDORT v3.6 Radiative Transfer Model [14,15,16].

Monthly air pollutant concentrations in the Beijing–Tianjin–Hebei region from 2005 to 2017 were obtained using the “Zonal Statistics” tool. The analysis was performed using ArcMap 10.2. We considered the entire region and aggregated the air pollution levels monthly. Population weighting of air pollutant concentrations at the district/county level was used to calculate air pollution exposure for the entire region. The specific steps are as follows: First, calculate the weight of the population of the district/county to the total population of the region. Second, the air pollutant concentration of the district/county is multiplied by the weight obtained in the previous step. Finally, the population-weighted air pollution exposure of the study area is obtained by summing the counties and districts across the region.

**Figure 2:**
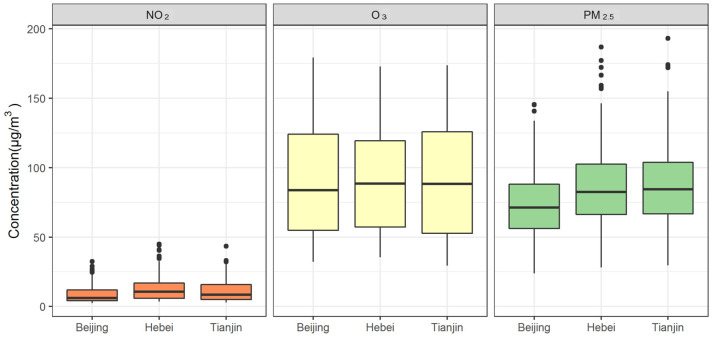
The distribution of the three air pollutant concentrations in Beijing, Tianjin and Hebei.

The distribution of the three air pollutants in Beijing, Tianjin and Hebei was relatively consistent (Figure 2), and the time series of air pollution concentrations in the three sub-regions also had high Pearson correlation coefficients (Table 1). It indicated that the spatial heterogeneity of air pollutant concentrations in Beijing–Tianjin–Hebei region was not strong, so air pollution averaged over the whole region was representative for the whole region.

Daily meteorological data were obtained from the China Surface Climate Information Daily Values Dataset (V3.0). We used the average values of the observations from 27 meteorological stations in the Beijing–Tianjin–Hebei region as the values of each meteorological factor in the region. Sunshine duration indicated the accumulation of actual sunshine hours for each day of the month. Precipitation represented the accumulation of rainfall for each day of the month. Relative humidity represented the ratio of absolute humidity in air to saturated absolute humidity at the same temperature and air pressure, and this study used the monthly average relative humidity. The monthly average values of temperature, wind speed and air pressure were used.

### 2.4. Socioeconomic Variables

Data regarding the per capita gross domestic product (PGDP), population density (PD), and number of medical technical personnel (MTP) from 2000 to 2015 were obtained from the China Statistical Yearbook. As they were found to be highly correlated, only PGDP was selected for inclusion in the model.

### 2.5. Statistical Analyses

Spearman’s rank correlation coefficients were used to explore the correlations between air pollutants and meteorological factors (Table 2). To avoid multi-collinearity, we included variables with correlation coefficients of <0.7 in the model.

The distributed lag nonlinear model (DLNM) is commonly used to investigate the health effects of air pollution and can describe associations by revealing potentially nonlinear and delayed effects for a time-series. This methodology is based on the definition of a “cross-basis”, a bi-dimensional space of functions that simultaneously describes the shape of the relationship along both the space of the predictor and the lag dimension of its occurrence [17].

In this study, we used a DLNM combined with Poisson regression to determine the nonlinear exposure–response relationship and lagged effects of PTB. Taking $Y_{t}$ as the number of PTB cases in the entire study area and assuming it follows a Poisson distribution, we obtain Equation (1):

(1) $$Y_{t}|\mu_{t}\sim Poisson\left(\mu_{t}\right)$$

where *t* is the number of months of observation, $Y_{t}$ and $\mu_{t}$ represent the actual and expected numbers of PTB cases in *t* months, respectively.

The single-pollutant model is as follows:

(2) $$Log\left(\mu_{t}\right)=\alpha +W_{X}^{T}\eta +ns\left(time,df\times year\right)+ns\left(RHU,3\right)+ns\left(SSD,3\right)\;+\eta \times Days+\gamma \times Optimization+\beta \times PGDP\;=\alpha +W_{X}^{T}\eta +COVs$$

where α is the intercept and $W_{X}^{T}\eta$ represents the cross-basis matrix of each pollutant. We adopted a linear and a natural cubic spline function to fit the exposure–response relationship and the response relationships between PTB cases and exposure to air pollutants [18]. A “*time*” variable (from 1 to 156 months) was used to control the long-term trends, and the degree of freedom (df) was set to “1 × *years*” by comparing Akaike information criterion (AIC) value, deviance of the model and the statistical significance of model parameters, the width of the confidence interval of estimated parameter [19]. Natural cubic splines with a priori 3 df were fitted to control meteorological variables as covariates [18]. Sunshine could reduce TB infection in the population by increasing the production of vitamin D in the human body, which plays a role in the host response to Mycobacterium tuberculosis [20]. And higher air humidity could cause *M. tuberculosis* to remain in the air longer, increasing the risk of tuberculosis infection. In addition, “*Days*” was the number of days per month. “*Optimization*” was used to control the impact of the TBMIS optimization and upgrade, which was a binary variable with a value of 0 in 2009 and earlier, and 1 in 2010 and later.

The progression from *M. tuberculosis* infection to clinically observed symptoms takes several months to years, and some latent infections can last a lifetime. Air pollution primarily causes *M. tuberculosis*, which is dormant in the human body and becomes active. In addition, the diagnosis and reporting of TB also require time, which can cause case finding delays. Leung et al. recommended a maximum case finding delay of 6 months [21]. Therefore, we assumed that it would take an average of 6 months for PTB cases activated by air pollution exposure to be diagnosed and recorded in the TBIMS, this period includes the manifestation of PTB, diagnosis and reporting; thus, we set the maximum lag time to 6 months.

The interim target levels in the Global Air Quality Guidelines (AQGs) of the World Health Organization (WHO) were used as reference data to calculate the effects of ambient air pollutant exposure on the number of PTB cases. The annual PM_2.5_ concentration in the Beijing–Tianjin–Hebei region is still within the interim targets 1 (IT-1) stage of the AQGs (2021); hence, we used a PM_2.5_ reference of 35 μg/m^3^. For O_3_ and NO_2_, the reference values were taken as 60 μg/m^3^ (peak season) and 10 μg/m^3^, respectively, as no targets are included in the AQGs (2021) for the monthly concentrations of O_3_ and NO_2_. Relative risk (RR) estimates and 95% confidence intervals (CI) were used to represent the lag-specific and cumulative risks of PTB cases for a 10-unit increase in the concentration of an air pollutant.

After modeling the single-pollutant regressions, we separately modeled the effects of air pollution on PTB cases by age group. Statistically significant differences in RRs with point estimates and 95% CIs between the <65 and ≥65 age groups were calculated as follows:

(3) $$\left(\hat{Q_{1}}-\hat{Q_{2}}\right)\pm 1.96\sqrt{{\left(\hat{SE_{1}}\right)}^{2}+{\left(\hat{SE_{2}}\right)}^{2}}$$

where $\hat{Q_{1}}$ and $\hat{Q_{2}}$ are the point estimates of the RRs for the two age categories, and $\hat{SE_{1}}$ and $\hat{SE_{2}}$ are their standard errors, respectively [22]. If the 95% CI contained a value of zero, there was no evidence showing modification by age [23].

We assessed the stability of the model by changing the df of calendar time (12–14 df) and meteorological factors (4–6 df) individually. All analyses were conducted using the “dlnm” and “splines” packages in R software (version 3.5.2). Statistical significance was set at *p* < 0.05.

## 3. Results

### 3.1. Descriptive Results

**Figure 3:**
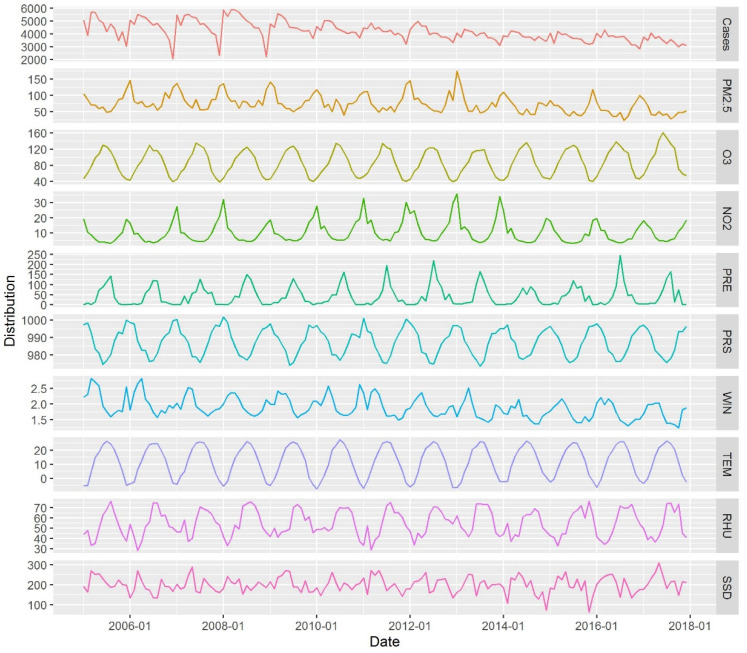
Monthly variations in the number of pulmonary tuberculosis (PTB) cases, air pollutant concentrations, and meteorological factors in Beijing–Tianjin–Hebei region.

From 2005 to 2017, 653,373 cases of PTB were recorded in the Beijing–Tianjin–Hebei region, 86.48% of which were people below 65 years of age and 13.52% were people aged 65 years or more. For cases involving people aged below 65 years, the highest number of cases were mainly observed in March and April of each year, whereas the lowest number of cases were observed in December. For cases involving people aged ≥65 years, the monthly maxima occurred in January and February in 6 of the 13 years considered. The monthly PTB cases exhibited a fluctuating downward trend and a noticeable seasonal pattern (Figure 3), with a high incidence in the spring and summer months. The mean monthly concentrations of PM_2.5_, NO_2_, and O_3_ were 87.72 μg/m^3^ (range of 28.28–178.24 μg/m^3^), 11.90 μg/m^3^ (3.45–41.66 μg/m^3^), and 89.41 μg/m^3^ (34.30–174.94 μg/m^3^), respectively (Table 3).

### 3.2. Effects of Air Pollution Exposure on the Risk of PTB

#### 3.2.1. PM_2.5_

**Figure 4:**
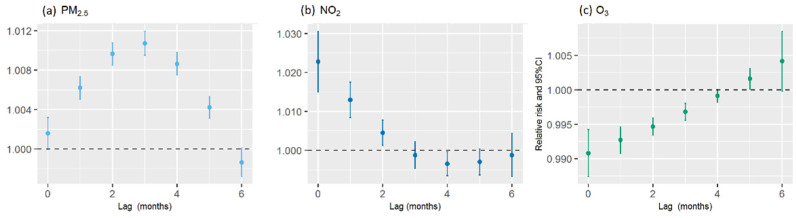
Lag-specific relative risks (%) of pulmonary tuberculosis (PTB) for 10-unit increases in the mean monthly concentrations of air pollutants based on single-pollutant models. (**a**) Air pollutant: PM_2.5_. (**b**) Air pollutant: NO_2_. (**c**) Air pollutant: O_3_.

**Figure 5:**
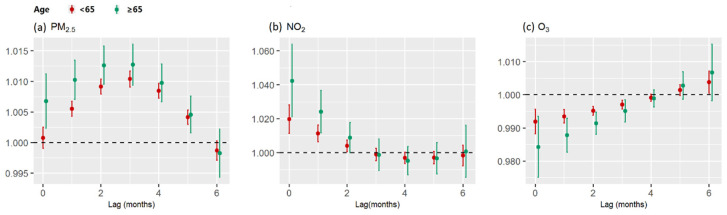
Lag-specific relative risks (95% confidence interval (CI)) of pulmonary tuberculosis (PTB) by age group for 10-unit increases in the mean monthly concentrations of air pollutants based on single-pollutant models. (**a**) Air pollutant: PM_2.5_. (**b**) Air pollutant: NO_2_. (**c**) Air pollutant: O_3_.

The RR–lag relationship of PM_2.5_ with reference to 35 μg/m^3^ is shown in Figure 4a. A 10 μg/m^3^ increase in the PM_2.5_ concentration was associated with an increased risk of PTB for a lag period of between 0 month (RR = 1.002, 95% CI: 1.000–1.003) and 5 months (RR = 1.004, 95% CI: 1.003–1.005). The RR increased gradually during a lag of 0–3 months, peaked at a lag of 3 months (RR = 1.011, 95% CI: 1.009–1.012), and then decreased to the lowest RR at a lag of 5 months. The cumulative RR (hereafter “cumRR”) peaked at a lag of 5 months (cumRR = 1.042, 95% CI: 1.036–1.048). Subgroup analyses revealed that the effect of PM_2.5_ exposure remained significant in people aged <65 years (cumRR = 1.039, 95% CI: 1.033–1.045, lag: 5 months) and people aged ≥65 years (cumRR = 1.058, 95% CI: 1.042–1.075, lag: 5 months), with no significant differences between the two age groups (Figure 5a).

#### 3.2.2. NO_2_

The RR–lag relationship of NO_2_ with reference to 10 μg/m^3^ is shown in Figure 4b. A 10 μg/m^3^ increase in the NO_2_ concentration was associated with an increased risk of PTB for a lag period of between 0 months (RR = 1.023, 95% CI: 1.015–1.031) and 2 months (RR = 1.004, 95% CI: 1.001–1.008). The RR showed a decreasing trend and decreased to 0 in the 3rd month. The cumRR was 1.041 (95% CI: 1.026–1.055, 2 months). Subgroup analyses revealed that the effect of NO_2_ exposure was significant in people aged < 65 years (cumRR = 1.035, 95% CI: 1.020–1.051, lag: 2 months) and people aged ≥ 65 years (cumRR = 1.077, 95% CI: 1.037–1.118, lag: 2 months), with no significant differences between the two age groups (Figure 5b).

#### 3.2.3. O_3_

A 10 μg/m^3^ increase in the O_3_ concentration was associated with a decreased risk of PTB for a lag period of between 0 month (RR = 0.991, 95% CI: 0.987–0.994) and 4 months (RR = 0.999, 95% CI: 0.998–1.000). The RR showed an increasing trend and rose to 0 in the fifth month (Figure 4c). The cumRR was 0.974 (95% CI: 0.968–0.981, 4 months). Subgroup analyses revealed that the effect of O_3_ exposure was significant in people aged < 65 years (cumRR = 0.977, 95%CI: 0.970–0.984, lag: 4 months) and people aged ≥ 65 years (cumRR = 0.958, 95%CI: 0.942–0.975, lag: 4 months), with no significant difference between the two age groups (Figure 5c).

## 4. Discussion

The Beijing–Tianjin–Hebei urban cluster is a cultural center in China with a high population density. Although the high level of air pollution in this region probably poses significant health risks, the relationship between air pollution exposure and the risk of PTB has not yet been investigated. Therefore, this study estimated the risk of developing PTB following exposure to three common pollutants (PM_2.5_, NO_2_, and O_3_) in the Beijing–Tianjin–Hebei region. We found that exposure to PM_2.5_ and NO_2_ was significantly associated with an increased risk of PTB, while exposure to O_3_ was associated with a decreased risk of PTB. In the subgroup analysis, no age-dependent effects were observed. The sensitivity results indicated that the model results were robust. After controlling other pollutants, the abovementioned relationships did not change despite changes in the RR values.

The results showed that exposure to PM_2.5_ led to an increased risk of PTB, with a lagged effect of up to five months. This finding is supported by the results of previous studies [4,5,24,25]. Exposure to PM_2.5_, may increase the risk of PTB in two ways: (1) by affecting the immune system response, and (2) by creating a lung environment conducive to the survival of *M. tuberculosis*. First, inhalation exposure to PM_2.5_ impairs important components of the protective human lung and systemic immune response against *M. tuberculosis* [26]. Studies have shown that exposure to PM_2.5_ could impair the immune function of anti-mycobacterial T cells [27,28]. Irrespective of seasonal variability, it has been observed that exposure to PM_2.5_ can decrease the release of pro-inflammatory cytokines and impair phagocytic functions of peripheral blood mononuclear cells in response to *M. tuberculosis* infection [29]. Sarkar et al. found that exposure to PM_2.5_ could increase the rate of developing TB and alter TB treatment outcomes [30]. Second, PM_2.5_ contains components that can promote the growth and reproduction of *M. tuberculosis*, such as transition metals [31,32]. In addition, due to its small diameter, PM_2.5_ can directly invade the human lungs, carrying harmful substances from the air into the bronchi and alveoli [6,7]. However, some studies are inconsistent with our findings [33,34]. Such inconsistencies may relate to confounder adjustment and differences in the methods used for exposure assessments. Therefore, the relationship between PM_2.5_ pollution and PTB should be further investigated.

In our analysis, we observed a significant statistical relationship between exposure to NO_2_ and the risk of PTB. The delay might be varied in different regions due to different environmental and social-economic factors. A study in London showed that the median case finding delays were approximately 3 months [35]. However, a study in China showed a case finding delay of 29 days (1 to 505d) for the Xicheng District of Beijing, China [36]. Another study showed patient delays and diagnosis delay of 15 and 10 days, respectively [37]. Since the Beijing–Tianjin–Hebei urban cluster is a political and cultural center in China with a high level of medical services, and the people living there are more aware of the importance of health screening, it might have a relative short case finding delay. Produced from combustion sources, such as motor vehicle exhaust and electricity generating units, NO_2_ is a gaseous pollutant that mainly irritates the lower respiratory tract and alveoli. Therefore, exposure to NO_2_ may help *M. tuberculosis* invade the alveoli and accelerate the progression of TB [7,33]. However, Liu et al. reported no significant relationship between NO_2_ and PTB [38].

We observed a significant negative association between O_3_ exposure and the risk of PTB, this agreeing with the findings of previous studies [7,33,39]. The lagged effect associated with O_3_ exposure reached 4 months and remained significant in both age groups. However, in a multi-city modeling study by Yao et al., an inverse correlation was observed between O_3_ exposure and PTB [40]. Moreover, most studies have found no evidence of a significant relationship between O_3_ exposure and TB outcomes; however, the findings were based on a limited sample size [40]. Thus, the relationship between O_3_ exposure and the risk of PTB remains controversial. In the present study, O_3_ exhibited a strong negative correlation with NO_2_. Individuals exposed to high NO_2_ concentrations are often exposed to low O_3_ concentrations [33,41], which could explain the inverse correlation between O_3_ exposure and the risk of PTB. Although O_3_ exposure may reduce the risk of PTB, it has other considerable adverse effects on the body. In experimental studies, O_3_ exposure in human alveolar macrophages was associated with decreased phagocytosis and impaired antimicrobial host defense [34,42,43]. Additionally, short-term exposure to high O_3_ concentrations can cause coughing, throat dryness, chest pain, increased mucosal secretion, fatigue, and nausea, which can significantly damage lung function, affect the structure of the respiratory tract, cause inflammation, alter airflow rates, and even lead to death [44]. The effects of O_3_ exposure should be interpreted with caution because the mechanisms of O_3_ in the human body are unclear and warrant further investigation.

Each air pollutant acts on the human body via different mechanisms and at different concentrations, and the curves of the lagged effects of exposure to the three pollutants with respect to the risk of PTB have their own patterns of variation. In this study, the lagged effects of PM_2.5_ exposure on the risk of PTB were highest in the third month of lagging, while the lagged effect of NO_2_ and O_3_ decreased gradually with an increase in the lag time. Exposures to high pollutant concentrations have gradual effects on the respiratory and immune systems; however, such effects depend on factors such as the fitness of the exposed individual, duration of exposure, and delay in diagnosis and reporting of PTB.

There were some limitations to our study, which was a population-based ecological study that did not involve individuals. First, we used data over a relatively long period, but could only consider a limited number of confounding factors. Second, although exposure to ambient air pollutants was correlated with the risk of PTB, we could not prove causality. Further research is needed to determine whether there is a true causal relationship. Third, the results of the study might be not generalizable; therefore, it would be prudent to extend to other areas.

## 5. Conclusions

The findings of this study showed that exposure to PM_2.5_ and NO_2_ was positively correlated with the risk of PTB, whereas exposure to O_3_ was associated with a decreased risk of PTB. No age-dependent effects were observed. Further research is required to explore the interactions between air pollutants and PTB, as well as the potential mechanisms. More attention should be given to the risks posed by air pollution. Active steps should be taken to reduce the concentrations of ambient air pollutants, which could reduce the spread of PTB and the associated health risks.

## Figures and Tables

**Table 1:** Pearson correlation coefficients of the time series of air pollution concentrations in Beijing, Tianjin and Hebei.

	NO_2_		PM_2.5_		O_3_	
	Beijing	Hebei	Beijing	Hebei	Beijing	Hebei
Hebei	0.96		0.89		0.98	
Tianjin	0.97	0.95	0.89	0.86	0.98	0.99

**Table 2:** Spearman correlation coefficients among ambient air pollutants and climatic factors.

	PM_2.5_	NO_2_	O_3_	PRE	PRS	WIN	TEM	RHU	SSD	PGDP	PD	MTP
PM_2.5_	1											
NO_2_	0.67 **	1										
O_3_	−0.68 **	−0.90 **	1									
PRE	−0.58 **	−0.85 **	0.79 **	1								
PRS	0.66 **	0.89 **	−0.94 **	−0.83 **	1							
WIN	0.24 **	0.30 **	−0.17 **	−0.45 **	0.19 **	1						
TEM	−0.67 **	−0.92 **	0.93 **	0.88 **	−0.94 **	−0.37	1					
RHU	−0.24 **	−0.51 **	0.38 **	0.74 **	−0.42 **	−0.82	0.57 **	1				
SSD	−0.39 **	−0.16 **	0.34 **	−0.05	−0.27 **	0.48	0.20 **	−0.50 **	1			
PGDP	−0.41 **	−0.02	0.06	0.02	−0.06	−0.36 **	0.03	−0.01	0.04	1		
PD	−0.41 **	−0.02	0.06	0.02	−0.06	−0.36 **	0.03	−0.01	0.04	0.99 **	1	
MTP	−0.41 **	−0.02	0.06	0.03	−0.06	−0.36 **	0.03	−0.01	0.04	0.99 **	0.99 **	1

Note: **: *p* < 0.01.

**Table 3:** Summary statistics of the number of pulmonary tuberculosis (PTB) cases, air pollutant concentrations, and meteorological factors in the Beijing–Tianjin–Hebei region.

Variables	Number (%)	Mean	SD		Percentile				
				Minimum	P25	P50	P75	Maximum	IQR
*Monthly recorded counts*									
Total	653,373 (100)	4188.29	747.28	2043.00	3722.00	4100.00	4656.50	5893.00	934.50
Age < 65	565,029 (86.48)	3621.98	640.44	1776.00	3220.50	3595.00	4034.00	5118.00	813.50
Age ≥ 65	88,344 (13.52)	566.31	118.24	267.00	481.00	546.50	643.25	962.00	162.25
*Atmospheric pollutants (μg/m^3^)*									
PM_2.5_	/	87.72	30.23	28.28	65.57	83.44	103.51	178.24	37.94
O_3_	/	89.41	36.61	34.30	54.44	87.27	121.75	174.94	67.30
NO_2_	/	11.90	8.27	3.45	5.66	9.62	15.50	41.66	9.84
*Meteorological factors*									
Precipitation (mm)	/	43.69	50.16	0.02	4.12	19.23	74.28	244.83	70.16
Air pressure (hPa)	/	987.39	7.78	973.48	980.16	988.09	993.61	1001.78	13.45
Relative humidity (%)	/	54.63	12.99	28.00	44.00	53.04	65.63	76.57	21.63
Temperature (°C)	/	11.63	11.14	−7.72	1.43	12.71	21.92	27.39	20.49
Sunshine duration (h)	/	200.95	40.45	63.53	176.59	200.65	229.36	310.57	52.77
Wind speed (m/s)	/	1.89	0.34	1.24	1.64	1.82	2.09	2.82	0.45

Note: IQR: Inter-Quartile Range; SD: Standard Deviation; PM: particulate matter.

## Data Availability

Pulmonary tuberculosis data is available at: https://www.phsciencedata.cn/Share/edtShareNew.jsp?id=39208 (accessed on 2 June 2021).

## Abbreviations

PTB	pulmonary tuberculosis
NO_2_	nitrogen dioxide
PM	particulate matter
O_3_	ozone
WIN	wind speed
TEM	temperature
RHU	relative humidity
SSD	sunshine duration
PGDP	per capita gross domestic product
PD	population density
MTP	numbers of medical technical personnel
IQR	Inter-Quartile Range
SD	Standard Deviation
